# Pair-matched analysis of Circulating Melanoma Cells (CMCs) before and after Immunotherapy in Relation to other Melanoma-Specific Biomarkers

**DOI:** 10.7150/jca.102131

**Published:** 2025-04-21

**Authors:** Paula Sawerska, Aneta Konwerska, Łukasz Galus, Agata Kolecka-Bednarczyk, Karolina Buszka, Damian Rusek, Agnieszka Seraszek-Jaros, Michał Nowicki, Jacek Mackiewicz, Joanna Budna-Tukan

**Affiliations:** 1 Department of Histology and Embryology, Poznan University of Medical Sciences, Poznan, Poland.; 2 Doctoral School, Poznan University of Medical Sciences, Poznan, Poland.; 3 Department of Medical and Experimental Oncology, Poznan University of Medical Sciences, Poznan, Poland.; 4 Department of Immunology, Poznan University of Medical Sciences, Poznan, Poland.; 5 Department of Forensic Medicine, Poznan University of Medical Sciences, Poznan, Poland.; 6 Department of Bioinformatics and Computational Biology, Poznan University of Medical Sciences, Poznan, Poland.; 7 Department of Anatomy and Histology, Collegium Medicum, University of Zielona Gora, Zielona Gora, Poland.

**Keywords:** melanoma, biomarkers, liquid biopsy, CMCs, immunotherapy

## Abstract

Melanoma remains challenging in terms of diagnosis and treatment, and there is an urgent need to implement accurate diagnostic methods and personalized treatment to improve clinical outcomes. Therefore, it may be useful to enrich the panel of melanoma markers already in use and develop combinations of biomarkers for disease prognosis and monitoring. Data suggest that a promising biomarker for such a combination is circulating melanoma cells (CMCs). Although the relevances of various biomarkers in diagnosis, prognosis and treatment monitoring in melanoma have been extensively studied, we aimed at comprehensive investigation and comparison of liquid biopsy and tissue biomarkers with clinical status of the patient. Specifically, we focused on CMCs, by comparing the number of CMCs, including pre- and post-treatment. Furthermore, we have assessed the expression of the PMEL and Melan-A markers and the S100B and TIMP-1 protein levels in representative blood samples from melanoma patients and healthy controls. The number of CMCs in the study group was significantly higher than in the CMC-negative control group. However, there was no significant difference between the incidence of CMCs in the pre- and post-treatment blood draws. Nonetheless, we have observed a negative correlation between LDH levels and PFS, and a negative correlation between S100B levels and lymphocyte counts. The results of the study indicate that combinations of biomarkers, rather than any single biomarker alone, possess the highest clinical application potential, which urges further research on larger patient groups.

## Introduction

Melanoma is a skin cancer predominantly affecting individuals with a large number of melanocytic lesions, a light skin phenotype, a positive family history, and high UV exposure 1. There is also some data regarding diagnosis and incidence in people with dark skin phototypes 2,3, in whom the cause seems to be not sun exposure-dependent. According to the American Cancer Society, between 2021 and 2022, the estimated number of new melanoma cases was lower than in previous years. This may be attributed to high public awareness of risk factors and methods of prevention publicized in educational campaigns. Nevertheless, even in 2022, the estimated number of deaths from melanoma reached 7,650, with the majority of them being men 4. Melanoma is, therefore, still a challenge in terms of diagnosis and treatment, and there is an urgent need for the implementation of accurate diagnostic methods and personalized treatment methods to improve the clinical outcome.

In choosing the best-fitting treatment method for a patient, the greatest clinical significance is attributed to prognostic methods used to assess the disease's course and monitor the therapy's effects 5. Markers currently used for clinical diagnosis, namely the serum levels of lactate dehydrogenase (LDH) and S100 calcium-binding protein B (S100B), exhibit several shortcomings, including lower sensitivity in the early stages of the disease 6. Similarly, the evaluation of premelanosome protein (PMEL) and melanoma antigen recognized by T cells 1 (Melan-A) expression, commonly used in melanoma diagnostics, carries the risk of ambiguity, as this marker can also be present on normal melanocytes 1,7.

The solution to this problem can lie in expanding the panel of already used melanoma markers and developing a new combination of biomarkers used for melanoma prognosis and monitoring. The available literature indicates that circulating tumor cells (CTCs), known in melanoma as circulating melanoma cells (CMCs), are a promising biomarker that could be used in such a combination 8. CMCs are tumor cells that, using mechanisms that enable mobility, detach from the primary tumor and, via the bloodstream, enter tissues vulnerable to metastasis formation 1. Data suggest that the number of CMCs in the bloodstream reflects the stage of the disease and susceptibility to metastasis formation. Association between CMCs, overall survival (OS), progression-free survival (PFS), LDH 9,10, and S100B levels 11 was also indicated. Interestingly, some reports describe the validity of using the tissue inhibitor of metalloproteinases 1 (TIMP-1) protein in melanoma research 12,13. This marker has already been associated with poor prognosis in many cancers 14, and revealing the association between TIMP-1 and other markers, especially CMCs, could be of great value.

To create a combination of CMCs with other melanoma markers, we need to determine the most accurate and least invasive method for detecting CMCs in patient blood. The CellSearch^®^ method, which has gained FDA approval for detecting CTCs in other types of cancer, seems particularly advantageous 15,16.

The main aim of this study was to explore the usefulness of the selected biomarkers in melanoma diagnostics, prognosis, and treatment monitoring with a particular focus on CMCs. This goal was completed using a comparison of CMC numbers, including their pre- and post-treatment status, evaluation of PMEL and Melan-A marker expression, and assessment of serum levels of S100B and TIMP-1 proteins in representative blood samples from melanoma patients and healthy controls. All results obtained were correlated and considered in relation to available patient clinical data.

## Materials and Methods

### Study design

Blood and paraffin-embedded tissue blocks were collected from qualified patients who gave their written consent for participation in the study. Three analyses were performed: a CMC count assessment from patients' whole blood using the CellSearch® system, determination of S100B and TIMP-1 proteins serum concentration using ELISA, and detection of PMEL and Melan-A expression on excised neoplastic lesions using immunohistochemical staining. Blood was collected twice, before the start of the treatment (baseline) and 2-4 months after the start of immunotherapy, to assess CMC counts and protein serum concentration. The obtained serum was aliquoted and frozen at -80˚C until testing.

The study was approved by the Bioethical Commeettee at Poznan University of Medical Sciences with resolution no. 451/20.

### Patient characteristics

The full study group consisted of 40 patients aged 37-88. All patients were adults with advanced, inoperable and/or metastatic cutaneous melanoma undergoing qualification for immunotherapy with anti-PD-1 antibodies (nivolumab or pembrolizumab) at the Department of Medical and Experimental Oncology, Heliodor Swiecicki University Hospital, Poznan University of Medical Sciences, Poznan, Poland, between 2019 and 2023. Patients were in the IV stage of the disease. The general condition of the patients was assessed as 0 or 1 according to the ECOG (Eastern Cooperative Oncology Group) performance scale. Patients with other concomitant cancer and significant automimmune diseases, as well as HBV, HCV and HIV infections and post-treatment complications were not eligible for the study.

The diagnosis was made based on the histopathologial result of the previously removed melanoma, its metastasis or biopsy. The molecular examination of the archived histopathological sample determined the mutation status in the *BRAF V600* gene. Before treatment, each patient underwent computed tomography of the head, chest, abdomen and pelvis, as well as panel of laboratory tests (morphology, ALAT, ASPAT, bilirubin, creatinine, amylase, electrolytes, glucose, TSH, fT4, LDH and general urine test) to determine the current advancement of the disease, organ function and possible contraindications to immunotherapy. The treatment was a standard therapeutic procedure that does not bear hallmarks of the medical experiment. The patients' response to the treatment, PFS and OS were assessed. Each patient gave oral and written consent to the proposed procedure.

The clinical characteristics of the patients include age (years), sex, stage of the cancer (I-IV), presence and location of metastases (M0-M1a-d), LDH serum level (U/l), lymphocyte count (10^9^/l), neutrophil count (10^9^/l), eosinophil count (10^9^/l), platelet count (10^9^/l), presence of *BRAF* mutation (1-positive, 0-negative), PFS (months), OS (months), ECOG (Eastern Cooperative Oncology Group; 0-5), ORR (Objective Response Rate; CR, PR, PD, SD, PD) and are presented in Supplementary Table S1.

Material from 20 patients was simultaneously analyzed using immunohistochemical staining.

The control group for CMC counts assessment and protein concentration evaluation consisted of 19 age-matched healthy individuals. Due to problematic acquisition of skin biopsies from the above-mentioned control group, controls for tissue expression determination consisted of 20 archival paraffin-embedded blocks of healthy skin biopsies.

### Assessment of circulating melanoma cells (CMCs)

The number of CMCs in patient and control blood samples was conducted using a fully automated CellSearch® system (CellTracks® Autoprep® System, CellTracks® Analyzer II® System, Silicon Biosystem, Menarini, Florence, Italy) and reagents developed specifically for this method (CellTracks Circulating Melanoma Cell Kit, Silicon Biosystem, Menarini, Florence, Italy, cat. no. CS0014). Blood was collected into 7.5 ml CellSave® preservation tubes (Silicon Biosystem, Menarini, Florence, Italy) and analyzed within a maximum of 24 h after collection. The analysis was carried out according to the manufacturer's procedure. The method employed magnetic beads (ferrofluid) coated with antibodies against the MCAM (CD146) antigen for CMC capture. Subsequently, enriched cells were immunostained with antibodies against HMW-MAA-PE (melanoma-associated antigen), CD34-APC (endothelial cell marker), CD45-APC (leukocyte marker), and DAPI (cell nucleus marker). Cells positive for MCAM and HMW-MAA and negative for CD34 and CD45 with intact nuclear signal were identified as CMCs. Results were presented as the number of detected CMCs in 7.5 ml of blood.

### Immunohistochemical staining and evaluation of PMEL and Melan-A proteins

Paraffin-embedded blocks with preserved tumor lesions were provided by the Department of Medical and Experimental Oncology, Heliodor Swiecicki University Hospital, Poznan University of Medical Sciences, Poznan, Poland. Twenty patients participated in this analysis and consented to the use of the material.

The material for the immunohistochemical test was fixed in 10% buffered formalin, embedded in paraffin, and cut on the microtome into 4-5 μm thick sections. Immunohistochemical reactions were performed on the previously prepared sections using the EnVision FLEX +, Mouse, High pH (Link) (Agilent Dako, Santa Clara, United States, cat. no. K800221-2). Heat-induced antigen demasking pre-treatment was also carried out using EnV FLEX TRS, Low pH (50 x) (Agilent Dako, Santa Clara, United States, cat. no. K800521-2). Then, the sections were incubated with anti-PMEL17 antibodies diluted 1:300 (Novus Biological, Bio-Techne Sp. z o.o., Warsaw, Poland, cat. no. NBP1-69571) anti-Melan-A antibodies diluted 1:300 (Novus Biological, Bio-Techne Sp. z o.o., Warsaw, Poland, cat. no. NBP1-30151) overnight at +4 °C. The reaction was imaged with DAB-diaminobenzidine chromogen with the addition of hydrogen peroxide. The final stage of the reaction consisted of staining the cell nucleus with hematoxylin, tissue dehydration, and immersion in anhydrous histofluid solution. During immunocytochemical reactions, negative and positive controls were performed. The slides were scanned using the Grundium Ocus Digital Slide Scanner with Olympus X Line Objectives (Evident, Tokyo, Japan).

The stained specimens were subjected to histochemical evaluation by a pathologist. The intensity of melanoma cell staining was scored as 0 (negative), 1+ (mild), 2+ (moderate), or 3+ (strong). The distribution of cancer cells staining was scored as 0 (no positive cells), 1+ (less than 10% of stained cells), 2+ (10-50% of stained cells), 3+ (51-80% of stained cells) or 4+ (more than 80% of stained cells). An immunoreactivity score (IRS) was derived for each specimen by multiplying the intensity score by the distribution score. The IRS score was interpreted as negative (0-1), mild (2-3), moderate (4-8), or strongly positive (9-12).

For control purposes, 20 sections of healthy skin were stained and processed, respectively.

### Evaluation of S100B and TIMP-1 proteins' serum concentration

To obtain serum for determination of S100B and TIMP-1 biomarker concentration, blood was collected into 6.0 ml clot activator tubes and left undisturbed for 15 mins. Subequently clot was removed by centrifugation at 1000-2000 x g for 10 mins in a refrigerated centrifuge. Enzyme-linked immunosorbent assays (ELISA) were performed using ready-made, standardized kits for S100B (Human S100B ELISA, Merck Life Science, cat. no. EZHS100B-33K) and TIMP-1 (Human TIMP-1 Quantikine ELISA Kit, Bio-Techne Sp. z o.o., cat. no. DTM100), according to the manufacturers' procedures. Briefly, standards, serum samples, and controls were added to the wells in equal volumes. The plates were covered and incubated with the presence of a shaker at room temperature. After this time, wells were washed multiple times with a wash buffer, and a detection antibody solution was added to each well. The plates were then covered and incubated, followed by a washing step. Next, an enzyme solution was added to each well and incubated. After washing, the substrate solution was added to the same volume and incubated for a respective time, followed by the direct addition of a stop solution in an equal volume. Absorbance was measured at 450 and 590 nm for S100B and at 450, 540, and 570 nm for TIMP-1 immediately after preparation. Baseline and control samples were tested in triplicates. Results were presented in pg/ml for S100B and in ng/ml for TIMP-1.

### Statistical analysis

Statistical analyses were performed using Statistica (data analysis software system), version 13 (TIBCO Software Inc. 2017; www.tibco.com). Quantitative data are presented as range and median. All results were first verified by a normality test (Shapiro-Wilk test). Since the test confirmed a lack of normality, a non-parametric U Mann-Whitney test was used to compare the results between groups and objective response rate (ORR). The Wilcoxon test was used to compare CMCs in both blood draws. A Spearman rank correlation test was used to check the relationship between the selected variables. A p-value of less than 0.05 was considered significant.

## Results

### Circulating melanoma cell detection rate obtained using the CellSearch® system

**Figure 1:**
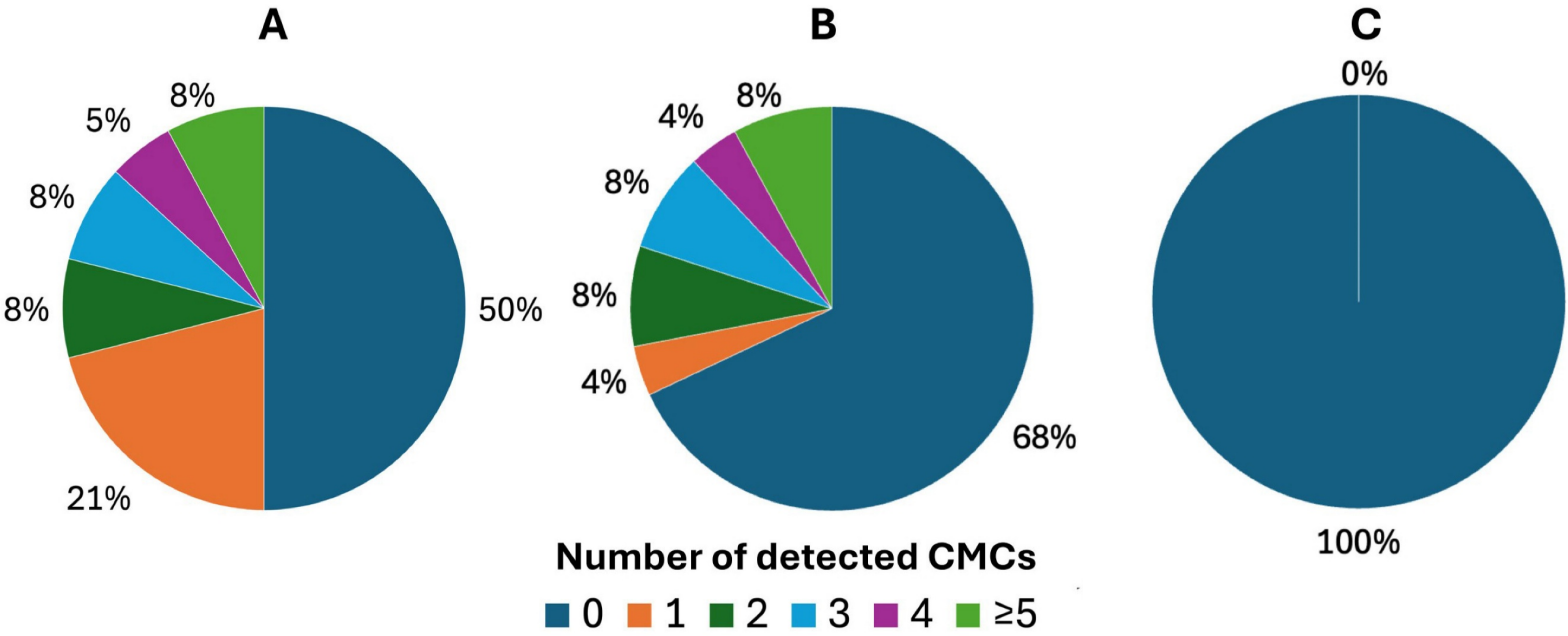
Distribution of detected circulating melanoma cells (CMCs) using the CellSearch® system tested separately before and after implemented therapy. Presented results of CMC enumeration show performance of technology in: A) melanoma patients before the treatment, B) melanoma patients after the treatment and C) control group of healthy individuals.

Two patients were excluded from the initial study group of 40 patients. One patient due to change in treatment regimen, the other one due to hemolysis of the sample, making counting of the cells impossible. Based on the CellSearch® system, we have demonstrated that 50% (19/38) of patients were CMC-positive during the first blood draw (range 0-8, median 0,5) (Figure 1A). CMC-positivity referred to ≥ 1 CMC detected in the blood sample. In case of the second blood draw, 25 samples were collected. This was caused by the inability to collect second blood sample from some patients due to logistic and clinical reasons, like death or change in treatment regimen. Among them, 32% (8/25) of patients were positive for CMCs (range 0-7, median 0) (Figure 1B). We found no CMCs (0 CMC) in the control group of healthy individuals (Figure 1C).

**Figure 2:**
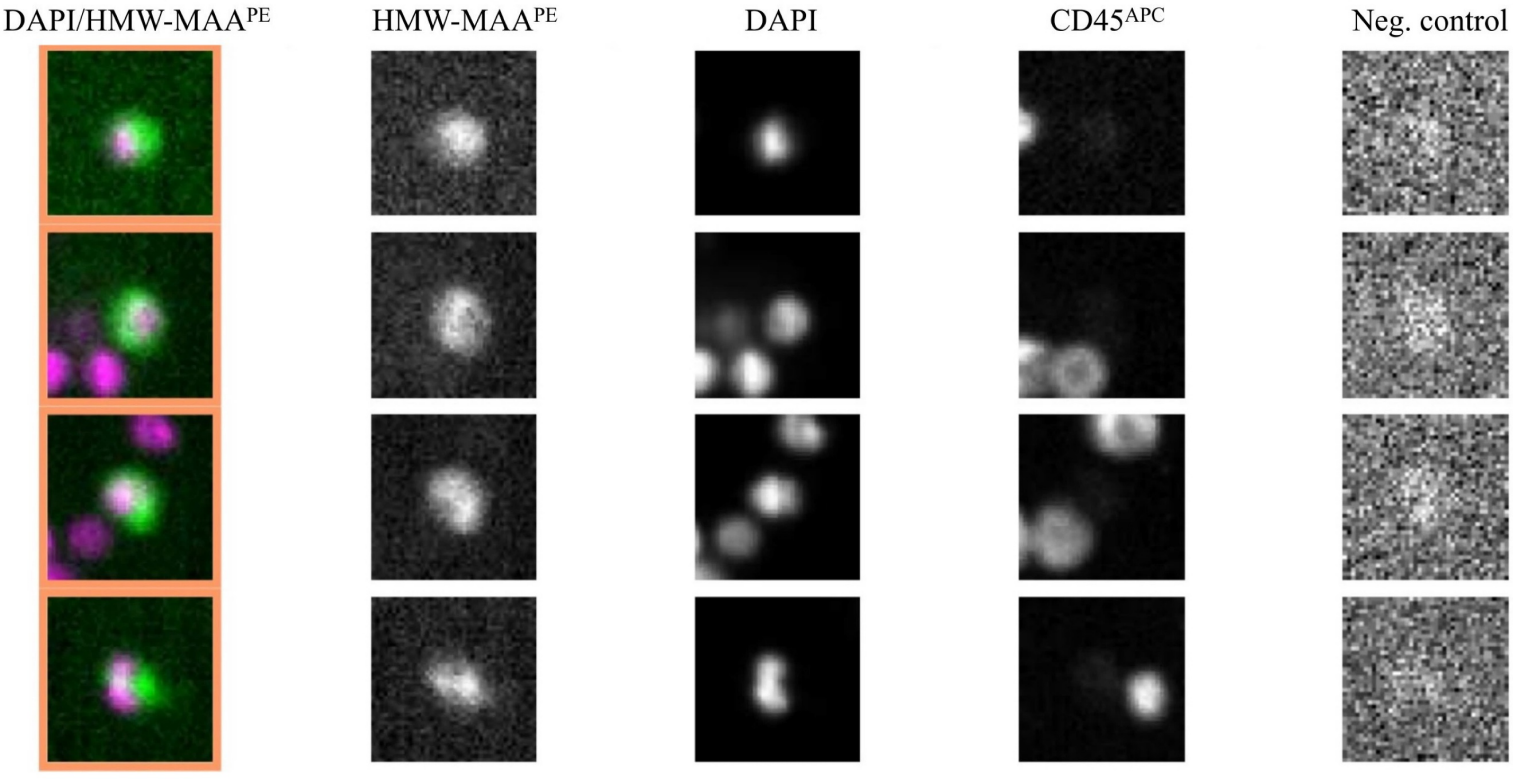
Representative images of CMCs detected in melanoma patients using the CellSearch® system. Enriched and stained cells were identified as tumor cells according to the following criteria: HMW-MAA-positive, DAPI-positive, CD45-negative, and negative for the last channel.

The difference between melanoma patients (baseline) and the control group was significant (p=0,0018). Representative images of results generated by the CellSearch® system are presented in Figure 2.

### Analysis of circulating melanoma cell counts in pair-matched blood samples before and after immunotherapy using the CellSearch® system

**Figure 3:**
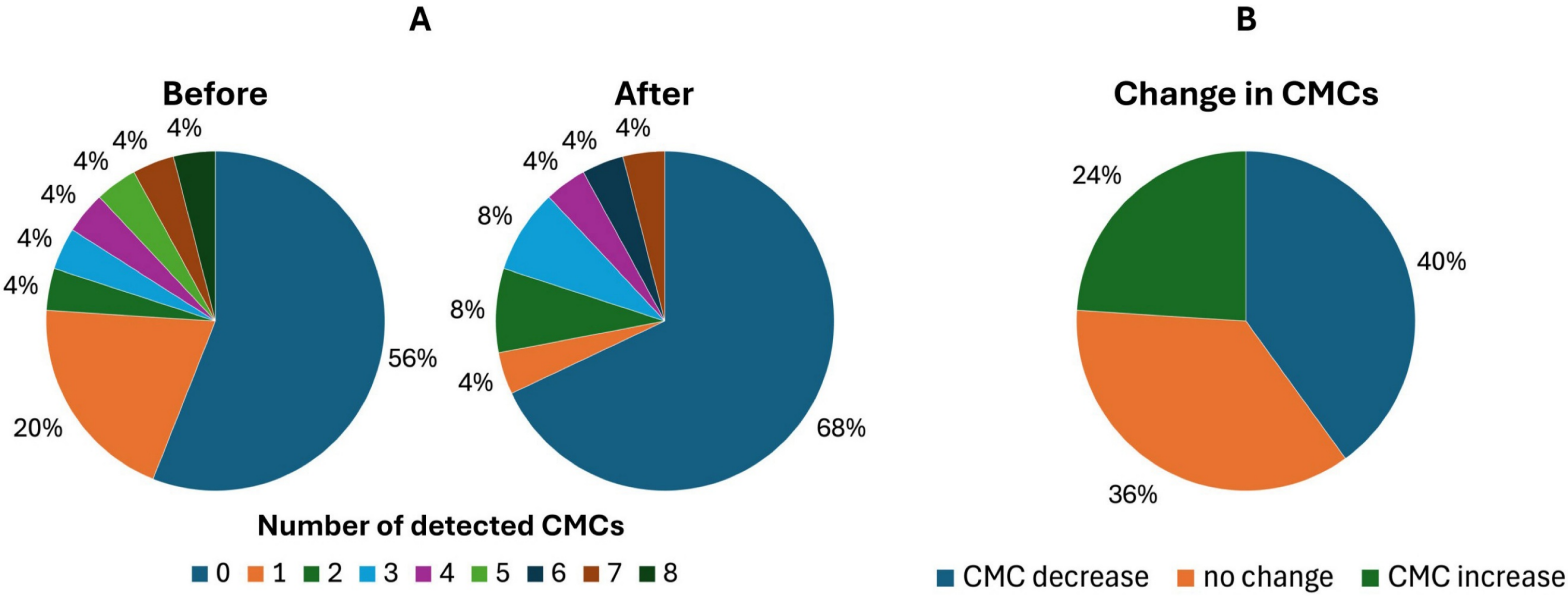
Circulating melanoma cells (CMCs) detection rate before and after treatment reflecting the effectivity of the implemented therapy. Matched-pair analysis of CMCs enumerated with the CellSearch® system: A) comparison of CMC counts in melanoma patients before and after the treatment, B) changes in CMC counts before vs. after the treatment.

We have found no statistically significant differences between the first and second blood draws (p = 0,7761). The matched CTC pairs were analyzed before and after immunotherapy for 25 patients (Figure 3A). In the post-treatment samples, the CTC positivity rate tended to decrease. Specifically, 44% (11/25) of patients were CTC-positive before and 32% (8/25) after treatment. CMC number decreased in 40% (10/25) of patients. The majority, 36% (9/25), showed no change, while 24% (6/25) exhibited an increase in the second analysis (Figure 3B).

### PMEL and Melan-A tissue expression

**Figure 4:**
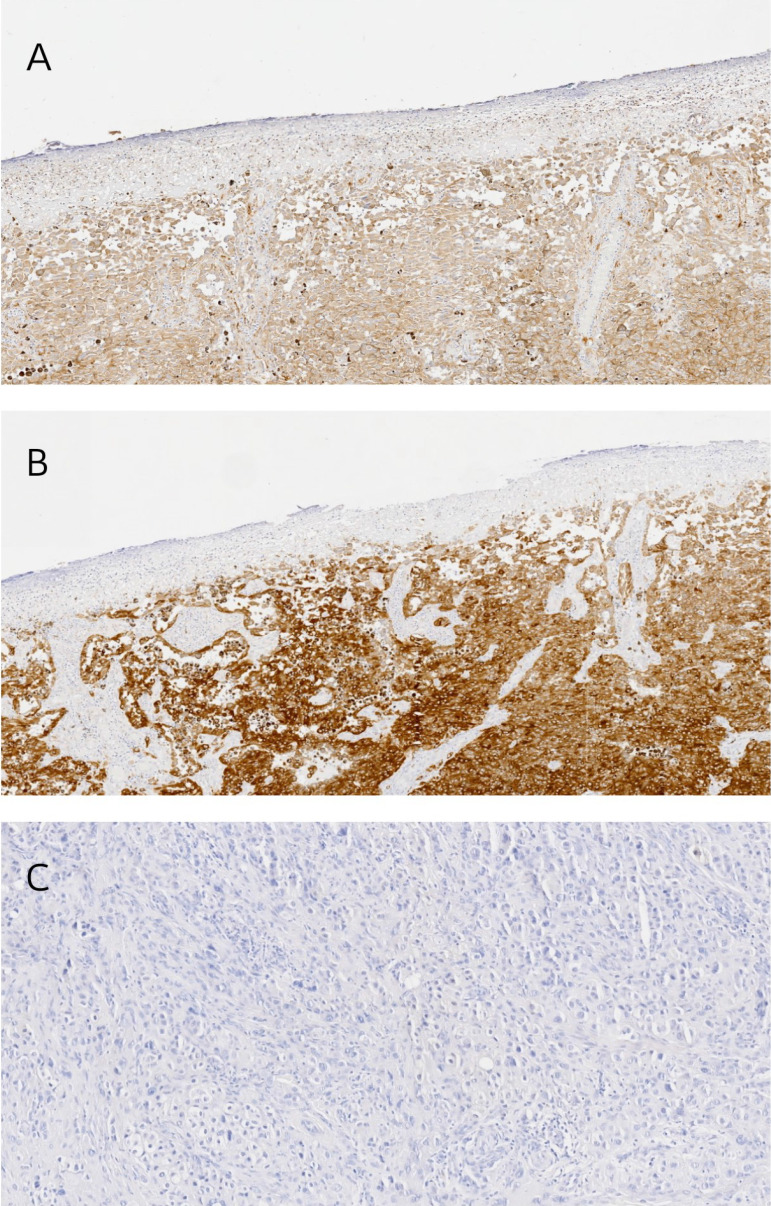
Representative images of immunohistochemical staining: A) positive PMEL expression, B) positive Melan-A expression and C) negative staining control.

From the initial study group of 40 patients, we were able to collect paraffin sections from 20. Most (n=19) of the analyzed lesions (n = 20) presented expression of both markers. In terms of PMEL expression, all patients were assessed as positive (Figure 4A). According to the IRS score, 4 were described as mild, 5 as moderate, and 11 as strongly positive. In the case of Melan-A, 19 of 20 patients presented expression (Figure 4B), with 1 of them classified as mild, 6 as moderate, and 12 as strongly positive. In both cases, the skin lesions examined are dominated by a high number of tumor cells. Negative staining control was presented in Figure 4C.

There was a significant difference between melanoma and melanocytes of healthy skin sections in the case of PMEL expression (p = 0,0073), but there was no difference when whole healthy skin was assessed (p = 0,1274). Surprisingly, healthy skin presented higher expression of PMEL than melanocytic lesions. On the other hand, there was no difference between melanoma and melanocytes of healthy skin sections in the case of Melan-A (p = 0,1143). Still, there was a difference when the whole, healthy skin was assessed (p < 0,0001), which presented a lower expression of Melan-A than melanocytic lesions.

### The concentration of S100B and TIMP-1 proteins in patients' serum

**Figure 5:**
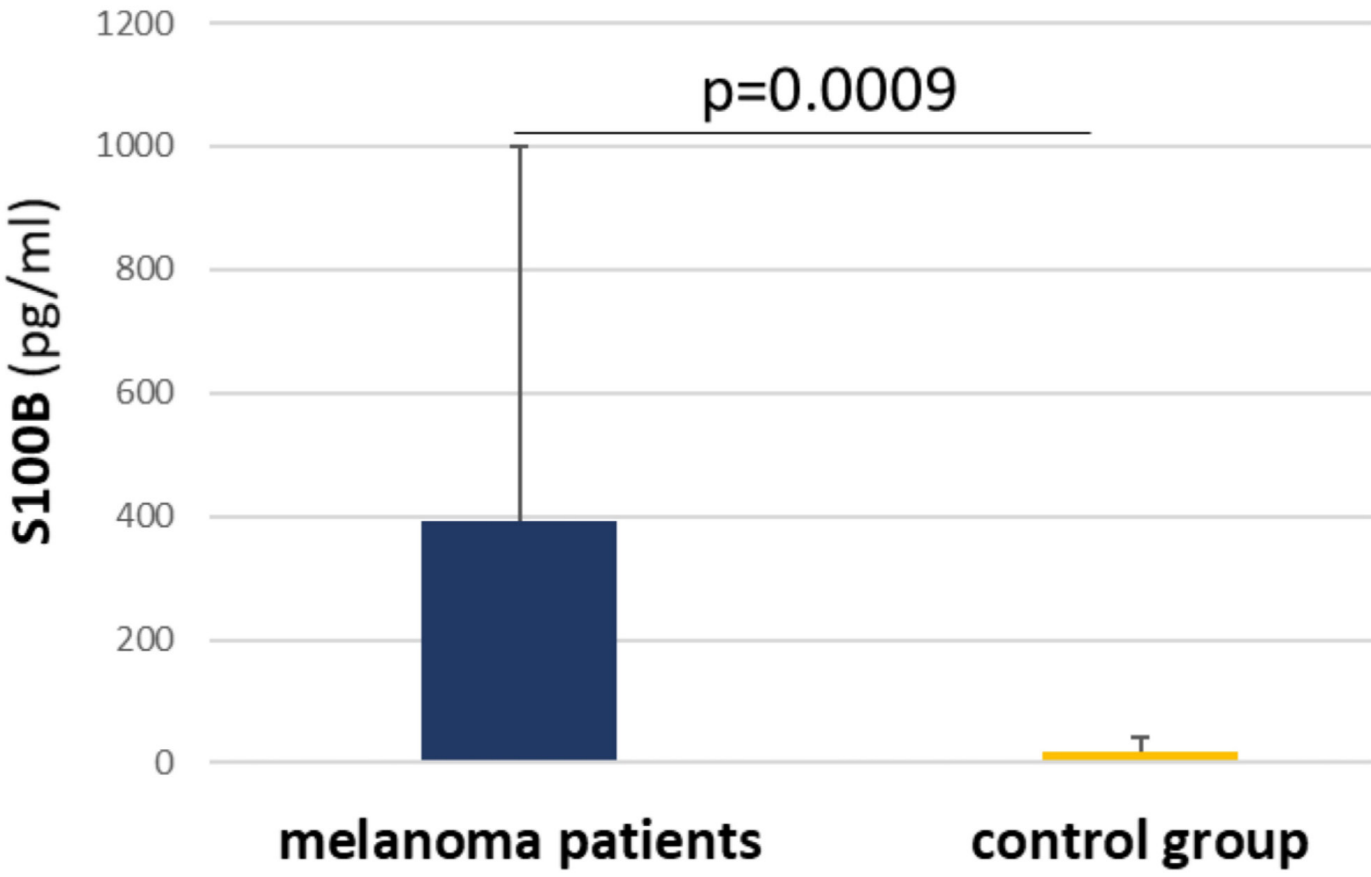
Bar graph illustrating significant differences in S100B concentration in serum between melanoma patient group and control group of healthy individuals.

Due to hemolysis, 5 patients were excluded from the S100B concentration measurement (n = 35). Patients' mean values oscillated between 2,759 pg/ml and 2394 pg/ml, with 5 patients classified as negative. In the control group (n = 14), mean values were between 7,336 pg/ml and 55,959 pg/ml, and 5 individuals were negative. Notably, there was a significant difference between the patient group, where the mean values were higher, and the control group (p = 0,0009) (Figure 5).

Regarding TIMP-1 concentrations (n = 35), 22 patients were positive and 13 negative. Mean values in the patients' group were between 0,9 ng/ml and 450,75 ng/ml. In the control group (n = 16), mean values were between 1,4 ng/ml and 232,5 ng/ml. Nine individuals were negative. We found no statistically significant differences between both groups (p = 0,7846).

### Correlation and relation between CMC count and selected parameters

Analysis showed a statistically significant negative correlation between the CMC count in the second blood draw and the lymphocyte count (R = -0.49, p = 0.0160), whereas no such a correlation was found as far as the CMCs from the first blood draw were concerned (R = -0.03, p = 0.8739). There was also no correlation between CMCs from both blood draws and other blood parameters, precisely: neutrophils (R = 0.11, p = 0.4999; R = -0,13, p = 0.5429 respectively), eosinophils (R = -0.16, p = 0.3784; R = 0.09, p = 0.6711 respectively) and platelets (R = 0.06, p = 0.7538; R = 0.22, p = 0.3111 respectively).

We did not obtain a correlation between the detected number of CMCs and OS in the first (R = 0.00, p = 0.9980) or second (R = 0.05, p = 0.8295) blood draw, as well as PFS in both blood draws (R = -0.01, p = 0.9659; R = -0.06, p = 0.7885 respectively). There was also no correlation between both CMC counts and LDH (R = 0.15, p = 0.9879; R = 0.25, p = 0.2322 respectively), S100B (R = -0.04, p = 0.2865; R = 0.00, p = 0.9978 respectively) and TIMP-1 (R = 0.14, p = 0.5773; R = 0.09, p = 0.6954 respectively) biomarkers, neither with Melan-A (R = -0.04, p = 0.8582; R = 0.02, p = 0.9471 respectively), PMEL IRS (R = 0.25, p = 0.2861; R = 0.28, p = 0.4276 respectively), the disease stage (R = 0.18, p = 0.2869; R = 0.21, p = 0.3164 respectively) and age (R = -0.05, p = 0.7552; R = -0.20, p = 0.3518 respectively).

Statistical analysis showed no relation between presence of *BRAF* mutation and CMCs from first (p = 0,6751) and second (p = 0,7675) blood draw as well as for presence or lack of metastasis in liver (p = 0,2863; p = 0,6419 respectively) or in CNS (p = 0,2863, p = 0,8362).

### Correlation between other melanoma-specific parameters

**Figure 6:**
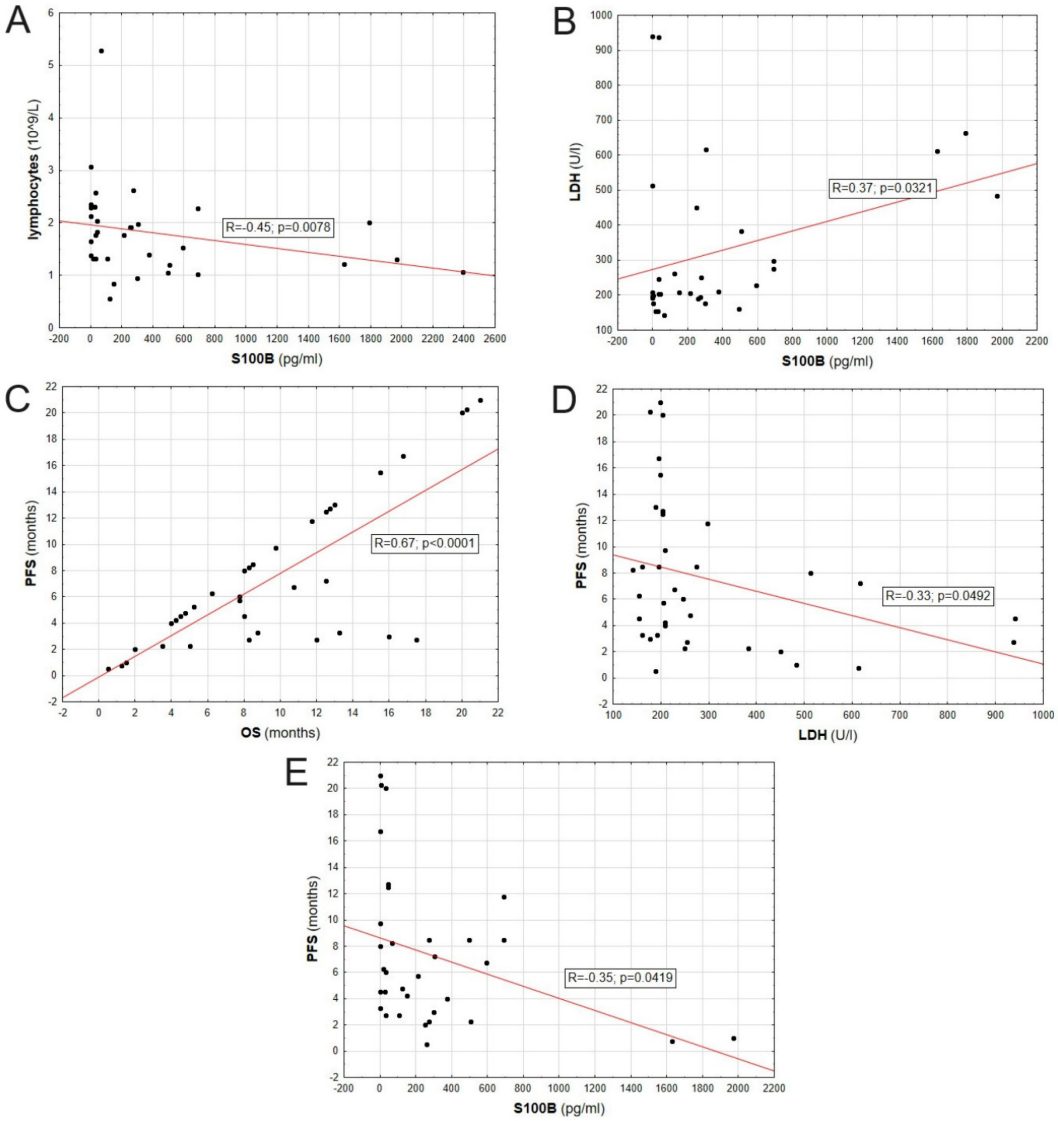
Correlation between parameters including: S100B serum concentration, LDH serum concentration, PFS, OS and lymphocyte count.

The lymphocyte count correlated negatively with the S100B protein level in patients' serum (R = -0,45; p = 0,0078). Also, LDH levels positively correlated with S100B protein level in patients' serum (R = 0,37; p = 0,0321). PFS positively correlated with OS (R = 0,67; p < 0,0001), and negatively correlated with LDH (R = 0,33; p = 0,0492) and S100B level (R = -0,35; p = 0,0419). Correlations between evaluated biomarkers are presented on Figure 6.

### Comperative analysis of the variables tested in relation to objective response rate (ORR)

We found statistically significant difference between PMEL IRS and ORR. The level of PMEL expression was lower in patients with partial response (PR) and complete response (CR), comparing to patients presenting stable disease (SD) and progressive disease (PD) (p = 0,031). A similar relationship was not observed for other parameters in relation to ORR: CMC count (p = 0,7907), Melan-A IRS (p = 0,3312), S100B (p = 0,2097), and TIMP-1 (p = 0,5605) serum concentrations.

## Discussion

Knowledge about circulating tumor cells has seen a continuous increase over the recent years. The presence and clinical utility of CTCs are already well described in the context of, e.g., prostate 17,18, breast 19, lung 20, and colorectal cancers 21. This prompts the researchers to study CTCs in other types of cancers, among others in melanoma 22. Palmieri et al. promoted CMCs detection for prognostic prediction in melanoma based on two assumptions. The first one, concerning the impact of cells detached from primary lesion on distant metastases formation, suggesting that enumeration of CMCs would help in detection of putative early metastatic spread. The second one, showing that although the presence of CMCs in the blood stream is not unequivocal indication of metastatic colonization, there are some promising results proving prognostic value of the CMC occurrence in the initial stages of the disease 23.

In fact, the mere presence of these cells in the blood is associated with a poor prognosis 24, and this risk increases with the number of cells detected 25,26. Their prognostic value has been described in a number of scientific publications, proving that the data collected before and after treatment, e.g., chemotherapy, immunotherapy, or other targeted therapy, can be used to monitor patient outcomes 27-29. Combining melanoma biomarkers into a single effective panel, including CMCs and LDH, which is widely used in diagnostics, could be particularly effective 30. Moreover, the available sources also point out the advantages of using CTC-derived ctDNA in the diagnostics of other types of cancer 31.

High tumor heterogeneity, a known issue in melanoma, needs to be taken into account when developing methods to analyze CMCs 32, as well as functional importance of proteins facilitating tissue invasion and metastasis in relation to melanoma prognosis. Among others, increased expression of melanocyte-specific MCAM showed correlation with shorter DFS, higher mortality 33, poorer patient prognosis after therapy 34 and is already implemented in the standard protocol of the CellSearch® system (Menarini) 24. This method is approved by the Food and Drug Administration (FDA) and presents a CTC recovery rate of 88% 15,16. In addition, favorable results have been obtained in comparisons with other commonly used methods, such as the ISET 35. The advantages of the method, the absence of false-positive signals, and the prognostic significance of CMCs themselves are all significant arguments for their widespread use in treatment monitoring. In the current study, the number of CMCs in the study group was significantly higher compared to the control group, which was CMC-negative, indicating the absence of false positive signals.

In a multicancer study by Raut et al., 67% of patients showed a reduction in the number of CTCs from baseline as a result of treatment. Successively, 22% of patients showed an increase in the number of CTCs, and 11% of patients showed no change in their number. In several representative cases, a reduction in the number of CTCs from baseline indicated effective treatment 36. Similarly, results obtained by Mun Yee Ko et al., in a study based on longitudinal real-time CMC monitoring in esophageal cancer, revealed that their pre-surgery to post-surgery numbers could be an independent factor of poor prognosis and treatment efficacy in patients subjected to surgical treatment and those receiving neoadjuvant therapy 37.

In a study of several melanoma cases, Kiniwa et al. showed that the total CTC count in four of five patients with stage IV melanoma varied in response to treatment with a BRAF/MEK inhibitor, suggesting that CTC count has the potential to be used as a marker of treatment response in patients with advanced disease 5. Another study by Hong et al., conducted on a prospective cohort of 49 patients treated with immune checkpoint inhibitors, revealed that a decrease in CMC counts over 7 weeks of therapy correlated with a significant improvement in PFS 38. However, according to some authors, the use of CMCs in monitoring the targeted therapy's effects is still a subject of discussion. These controversies relate to the method of CMC analysis, differences in disease stages among study groups, and the frequency of blood sampling, making it nearly impossible to reach a consensus 35. The study of Susskind et al. is an example of such conflicting research, as no significant difference was found in the number of CMCs before and after various therapies, and the number of CMCs was not associated with the development of metastases at a short median follow-up time of 16 months 39. Our results stay in accordance with the results of this study, as we did not observe a statistically significant difference between the prevalence of CMCs in two sequential blood draws, pre- and post-treatment. However, after the treatment, the number of detected cells in the sample tended to decrease. Although all patients responded to the treatment applied, some of them showed progression in subsequent examinations, with one developing autoimmune hepatitis. It is possible that this may have affected the obtained results, and the abundance of CMCs still can indicate the effectiveness of the treatment applied and predict the course of the disease.

All the problems related to CMC rarity and nonstandardized methods of their detection still result in the ambiguous relevance of CMCs in the diagnosis and treatment monitoring of melanoma patients. Each ongoing and completed study brings new data and deepens our knowledge and understanding of this challenging field. Taking this into account, it is crucial to look for relations between CMCs, well-known melanoma biomarkers, and patient's clinical data. Among others, LDH and S100B serum levels, as well as OS and PFS, are highly promising.

We did not observe a correlation between the detected number of CMCs, OS, PFS, and biomarkers like LDH and S100B, although the work of other researchers suggests that such a relationship could potentially occur. LDH is a strong prognostic marker in metastatic melanoma, and its increased levels correlate with decreased survival in advanced-stage patients 40. The results of Cayrefourcq et al., also based on the CellSearch® method, indicate that LDH was significantly correlated with CMC detection 11. The same study found a significant association between OS and CMC count ≥2 per 7.5 mL of blood 11. Similar results were obtained by Rao et al, indicating that OS was shorter in patients with ≥2 CMCs per 7.5 mL of blood 16. In turn, Khoja et al. demonstrated that median OS was significantly shorter in patients with ≥2 CMCs detected in a blood sample 41. Hall et al. used adjusted Cox models and found a significant association suggesting worse PFS at 180 days in patients who were CTC-positive at the start of the study (compared to CTC-negative). One or more CTCs detected at the start of the study were associated with progression within 180 days in patients with stage IV melanoma 42. Furthermore, Hida et al. showed that CMCs could classify patients with poor prognosis. They noted that the median survival time for the patients with < 2 CMC was 19.5 months, and for those with ≥ 2 CMCs - 4.5 months. Moreover, they confirmed that the CellSearch® system is a standardized, reproducible, and useful tool in cancer studies 43. Li et al. indicated that measuring baseline and post-treatment CMC count is a powerful approach for monitoring melanoma progression, response to treatment, and survival. In their publication, a high baseline CMC count was correlated with short OS and was found to be an independent prognostic factor. Moreover, a change in CMCs pre- and post-treatment was an indicator of PFS and disease-specific survival (DSS). However, the authors did not find a significant association between the number of CTCs and the histological type of tumor, gender, age, and the S100B level (which has been confirmed by our results) 9.

Expression of the S100B protein was already found to positively correlate with the presence of metastases, prognosis and survival 11. While S100B is not suitable as a marker in the early stages of the disease, as it is sometimes undetectable in serum, study data indicate its prognostic value in higher stages of melanoma. The protein is released into the circulatory system as a result of loss of cell integrity, and proteolytic degradation associated with apoptosis. In addition, data suggest its usefulness in therapy monitoring, effective in both chemotherapy and immunotherapy 44. A meta-analysis by Janka et al. suggests that serum S100B concentration is important in the prediction of disease recurrence 45. In the current study, we found a significant difference in S100B concentration between the study and control group, although we have not found a significant correlation between S100B and CMC counts. Interestingly, we have detected an association between S100B concentration and LDH concentration in patients' serum, PFS, and OS. Felix et al. proved that lower levels of S100B and LDH correlated with better response and survival 46. A correlation between S100B and LDH was also demonstrated in the publication of Deckers et al. Elevated LDH was described as the independent prognostic factor for survival. The authors have shown that S100B and LDH appear to indicate different aspects of metastatic disease 47. On the other hand, in an analysis conducted by Karonidis et al., no significant association with LDH was found. It was shown that serum S100B levels reflect tumor burden, correlate with treatment response, and might be useful in identifying the risk of recurrence. Furthermore, S100B > 0.5 μg/l was associated with stage IV and poor survival 48.

The literature also points to evidence suggesting a potential negative correlation between S100B levels, OS, and PFS in melanoma patients, particularly in those with advanced or metastatic disease. Elevated S100B levels were previously associated with poor prognosis and shorter survival. An S100B study conducted by Tarhini et al. showed that higher levels of S100B were associated with a higher risk of recurrence and death. Moreover, a baseline S100B level ≥0.15 μg was significantly correlated with OS 49. According to Harpio et al. survival is significantly longer in melanoma patients with normal S100B levels compared to those with elevated levels of this protein. Furthermore, S100B levels reflect tumor mass, with serum levels of this marker predicting treatment effectiveness. A decrease in the measured S100B levels is associated with response to treatment, while progression is associated with increased concentration of this protein 50. In a study by Hauschild et al., the estimated OS time was significantly longer in patients with S100B values below 0.2 μg/l compared to patients with elevated S100B levels (≥ 0.2 μg/l), indicating the values as an independent indicator of disease stage (I-IV) 51. Moreover, in a meta-analysis by Mocellin et al. that studied the overall association between serum S100B levels and patient survival, elevated serum S100B concentration was associated with significantly worse survival. However, the author noted a significant heterogeneity between study designs and results 10. It is important to note that the relationship between S100B levels and survival outcomes might be influenced by various factors, including tumor stage, treatment modalities, and patient characteristics. Gebhardt et al. indicated that false-positive results lead to anxiety among patients and increase the number of costly diagnostic procedures, suggesting a necessity to analyze cases of excessive S100B release. The author noted that false-positive results may be related to comorbidities and require careful reassessment 52.

We also detected a negative correlation between LDH levels and PFS. Many authors presented similar results, even in the context of different types of cancer. For instance, in a study by Faloppi et al. LDH was found to be an indirect marker of neoangiogenesis and a worse prognosis. An association between LDH values and median OS and PFS was noted by the author 53. Moreover, Zhang et al. demonstrated that high LDH expression was significantly correlated with worse OS in urologic cancer. High serum LDH levels were associated with OS and PFS in patients with urologic cancer and were indicated as an effective biomarker of prognosis 54. In turn, in the study by Li et al., higher LDH values were significantly correlated with shorter median PFS and OS 55. Moreover, analyses by Capone et al. showed that an increase in LDH was significantly associated with short OS 56.

We have also found a negative correlation between S100B level and lymphocyte count. While both factors can provide important information about the status of melanoma and the immune response, their correlation in melanoma patients has not been well-established. It is possible that, in some cases, as melanoma progresses and S100B levels increase, a decrease in lymphocyte counts can occur due to the suppression of the immune system by the cancer. However, this relationship may not hold true in all cases and can vary depending on individual patient characteristics and other factors 40,57.

Except for S100B, the serum concentration of TIMP-1 is perceived as another promising biomarker in melanoma 58,59. In a study by Kluger et al., using similar methods, TIMP-1 levels were shown to be higher in patients with stage IV melanoma, compared to healthy controls and patients with thin primary melanomas 58. Similarly, Lugowska et al. found that TIMP-1 levels were significantly higher in the patient group than in the control group. Additionally, elevated TIMP-1 levels at the time of diagnosis were associated with a lower percentage of 3-year disease-free (61% vs. 81%) and 3-year overall (82% vs. 88%) survivors 60. Yoshino et al. demonstrated that serum TIMP-1 levels differ between the patient group and the control group. While they were higher in the patient group, there were notable differences between the patients themselves. Moreover, at stage 4, the levels were higher than at the lower stages. The authors also looked at post-mortem levels, which were higher than in the living patients 59. The above observations were also confirmed on a cellular level. Toricelli et al. described TIMP-1 relevance in melanocytic anoikis, resistance along with β1-integrin, and CD63 expression 12. A broadened study of this group revealed that TIMP-1 promotes melanoma cell survival by activating the PDK1 signaling pathway, and enhances resistance to anoikis through simultaneous activity with PKC, especially in advanced tumor stages 12. Similarly, Hoashi et al., found out that elevated TIMP-1 levels were associated with the raised rate of cell migration and promoted growth of primary melanoma cell lines 61. The utility of TIMP-1 tissue expression was also described in other types of cancers 62, including breast cancer 63, underlying novel perspectives for therapeutic approaches. Unfortunately, we did not find any correlations or associations to support this theory. Furthermore, we did not find any differences in TIMP-1 expression between the study group and the control group. Perhaps the results should be extended, and other test material from patients, such as serum, should be investigated. Dresse et al. suggested that TIMP-1 is unstable and needs to be carefully assessed, with blood centrifugation immediately after venipuncture 64, which is rarely possible in a clinical setting.

Finally, tissue biomarkers were found promissing prognosticators based on series of independent studies evaluating their individual and synergistic effect on supporting tumor progression, however evaluation on the same cohort and sophisticated statistical tools are required to draw final conclusions 33. PMEL, considered to be an indicator of melanoma 65, was found to be overexpressed at all stages of melanoma progression and was indicated as a specific marker of melanoma with low expression in other tissues 7. This seems to be confirmed in our study, where all patients were PMEL-positive, with half of them presenting strong expression. Most importantly, our analysis revealed that level of PMEL expression was lower in patients with partial response (PR) and complete response (CR), comparing to patients presenting stable disease (SD) and progressive disease (PD) after treatment. This strongly suggests that PMEL is a promising response biomarker. In addition, the majority of subjects showed simultaneous expression of Melan-A, which, similarly to PMEL, is considered a typical melanoma marker 66. However, there are numerous publications suggesting that its expression is not necessarily associated with melanoma or other cancers 67, which was confirmed by our study on healthy skin sections, where the expression was also noted. Danga et al. noted that the absence of cytological atypia in Melan-A-expressing cells and their presence in resected non-melanocytic tumors supports the idea that these are benign, reactive melanocytes 68. Furthermore, Orosz Z claimed that in desmoplastic melanoma, variable Melan-A staining requires detailed histological examination and complementary S100 staining 69, which were found to be mutually correlated 70.

To summarize, some connections between distinct biomarkers and the clinical status of melanoma patients were observed. However, they were rarely straightforward and repeatable, indicating that their complex involvement in clinical outcomes requires a deeper analysis on a large and homogenous group of patients in diverse stages of the disease. Taking into account the already proven utility of CTCs and the constant improvement of their detection rates and characterization methods, approaches based on these cells could become a staple in the future of melanoma diagnostics. Nonetheless, the results of this study and the available literature all suggest that it is not the selection of a single biomarker but rather a combination of the most sensitive and specific markers that would be of the greatest value. This urges further research on this topic.

## Supplementary Material

Supplementary table.
