# Supplementary material for: Pair-matched analysis of Circulating Melanoma Cells (CMCs) before and after Immunotherapy in Relation to other Melanoma-Specific Biomarkers
Source: J Cancer. 2025 Apr 21;16(8):2421–33. doi: 10.7150/jca.102131 (PMC12170499; doi:10.7150/jca.102131)
Supplement: Supplementary file 1 — Supplementary table. [file jcav16p2421s1.pdf]

Supplementary materials

Table S1. Clinical characteristics of patients participated in the study (n=40).

Patient - code	Age (years)	Sex	Stage	LDH (U/L)	Lymphocytes count (10^9/L)	Neutrophils count (10^9/L)	Eosinophils count (10^9/L)	Platelets count (10^9/L)	BRAF mutation (1- positive)	PFS (months)	OS (months)	ECOG score (0-5)	ORR
**1**	79	F	IV, M1a0	198	2,29	7,86	0,48	260	1	21	21	1	PR
**2**	56	F	Excluded from the study due to change in treatment regimen.										
**3**	70	F	IV, M1a0	177	1,64	4,79	0,03	289	1	20,25	20,25	0	PR
**4**	57	F	IV, M1a1	229	1,52	3,8	0,19	3	0	6,75	10,75	0	SD
**5**	85	F	IV, M1a0	196	2,35	3,72	0,14	254	0	16,75	16,75	1	PR
**6**	73	M	IV, M1b0	203	2,58	7,03	0,25	287	1	20	20	1	SD
**7**	62	M	IV, M1d1	938	1,32	6,6	0,1	230	1	2,75	17,5	1	PD
**8**	62	M	IV, M1b0	189	1,92	5,35	0,1	247	0	0,5	0,5	0	PD
**9**	62	M	IV, M1c1	484	1,3	8,81	0,04	222	1	1	1,5	1	PD
**10**	38	M	IV, M1a0	177	0,94	3,38	0,09	248	1	3	16	1	PD
**11**	49	M	IV, M1d0	198	1,31	6,73	0,03	269	1	15,5	15,5	0	PR
**12**	82	F	IV, M1a0	208	1,38	10,03	0,24	306	0	9,75	9,75	1	PR
**13**	71	M	IV, M1a0	161	2,8	5,25	0,28	231	0	3,25	13,25	1	PD
**14**	76	M	IV, M1d0	154	1,32	7,62	0,19	412	1	6,25	6,25	1	PR
**15**	58	F	IV, M1d1	383	1,2	10,54	0	440	0	2,25	3,5	1	PD
**16**	69	M	IV, M1a0	189	2,01	3,9	0,06	262	1	13	13	0	PR
**17**	63	F	IV, M1a0	203	1,83	5,02	0,06	371	1	12,75	12,75	0	CR
**18**	57	F	IV, M1d1	613	1,21	5,19	0,04	585	1	0,75	1,25	2	PD
**19**	62	F	IV, M1c1	616	1,97	3,4	0,65	411	1	7,25	12,5	0	PR
**20**	79	M	IV, M1a0	203	2,03	3,61	0,16	278	1	12,5	12,5	1	PR
**21**	73	M	IV, M1a1	255	1,11	4,73	0,26	182	0	2,75	12	1	PD
**22**	68	F	IV, M1b0	155	2,3	3,23	0,6	262	0	4,5	4,5	1	CR
**23**	84	M	IV, M1a1	450	1,92	8,96	0,08	542	0	2	2	1	-
**24**	61	F	IV, M1a1	297	1,02	5,12	0,19	99	1	11,75	11,75	1	PR
**25**	69	M	IV, M1c0	192	2,33	4,25	0,18	221	0	3,25	8,75	0	PD
**26**	62	F	IV, M1b0	209	1,4	2,89	0,16	191	1	4	4	0	PR
**27**	66	F	IV, M1c0	161	1,05	1,95	0,13	357	0	8,5	8,5	0	PR
**28**	75	F	IV, M1a0	195	N/A	bd	bd	n/a	0	8,5	8,5	1	PR
**29**	88	F	IV, M1a1	274	2,27	1,74	0,09	225	0	8,5	8,5	1	PR
**30**	59	F	IV, M1a0	142	5,28	3,64	0,14	268	0	8,25	8,25	1	CR

**31**	47	M	IV, M1c0	bd	1,32	2,41	0,17	322	1	2,75	8,25	0	PD
**32**	60	F	IV, M1d1	940	3,07	4,31	0,17	172	1	4,5	8	0	SD
**33**	44	M	IV, M1d1	513	2,12	4,16	0,06	265	1	8	8	0	PR
**34**	76	M	IV, M1a0	247	1,76	2,13	0,36	278	0	6	7,75	0	PR
**35**	49	M	IV, M1d0	205	1,77	4,34	0,16	285	0	5,75	7,75	0	PR
**36**	79	M	IV, M1a1	262	0,55	8,63	0,07	223	0	4,75	4,75	1	CR
**37**	42	F	IV, M1d1	2423	1,07	12,13	n/a	n/a	1	5,25	5,25	0	PR
**38**	62	F	IV, M1b1	664	2	4,15	n/a	n/a	1	-	5	1	PD
**39**	59	F	IV, M1d1	250	2,62	6,09	n/a	n/a	0	2,25	5	0	PD
**40**	74	M	IV, M1c0	208	0,84	5,32	n/a	n/a	1	4,25	4,25	1	PR

M – male; F – female; M1a - metastases in the skin, subcutaneous tissue, or lymph nodes; M1b - lung metastases without M1a; M1c - metastases in other organs without M1a and M1b, except metastases in the central nervous system; M1d - metastases in the central nervous system without M1a, M1b, and M1c; LDH - lactate dehydrogenase; PFS - progression-free survival; OS - overall survival; ECOG score - Eastern Cooperative Oncology Group performance score; ORR - objective response rate; PR - Partial Response; SD - Stable Disease; CR - Complete Response; PD - Progressive Disease.
